# Experimental and Natural Infections of Tick-Borne Encephalitis Virus in Dogs

**DOI:** 10.3390/v13102039

**Published:** 2021-10-09

**Authors:** Jiri Salat, Milan Hunady, Pavel Schanilec, Petra Strakova, Michal Stefanik, Pavel Svoboda, Lucie Strelcova, Jana Bojcukova, Martin Palus, Daniel Růžek

**Affiliations:** 1 Laboratory of Emerging Viral Infections, Veterinary Research Institute, Hudcova 70, 62100 Brno, Czech Republic; strakova.p@centrum.cz (P.S.); michal.stefanik2@gmail.com (M.S.); svoboda@vri.cz (P.S.); jsirmarova@gmail.com (J.B.); palus@paru.cas.cz (M.P.); 2 Biology Centre of the Czech Academy of Sciences, Laboratory of Arbovirology, Institute of Parasitology, Branisovska 31, 37005 Ceske Budejovice, Czech Republic; 3 Bioveta, Inc., Komenského 212, 68323 Ivanovice na Hane, Czech Republic; hunady.milan@bioveta.cz (M.H.); strelcova.lucie@bioveta.cz (L.S.); 4 Faculty of Veterinary Medicine, University of Veterinary Sciences Brno, Palackeho tr. 1946/1, 61242 Brno, Czech Republic; Schanilecp@VFU.cz; 5 Department of Chemistry and Biochemistry, Mendel University in Brno, Zemedelska 1665, 61300 Brno, Czech Republic

**Keywords:** tick-borne encephalitis, dogs, experimental infection, seroprevalence, experimental infection

## Abstract

Dogs are frequently infected with the tick-borne encephalitis virus (TBEV). However, to date, only a few clinically manifest cases of tick-borne encephalitis (TBE) have been reported in dogs. In this study, three-month-old beagle dogs were infected with TBEV through a subcutaneous injection. Body temperature, clinical signs, blood haematology, blood biochemistry, and immune responses were monitored for up to 28 days postinfection (p.i.). No changes in body temperature or clinical signs were observed in the infected dogs. Most haematology and blood biochemistry parameters were unchanged after the infection, except for a slight reduction in blood lymphocyte counts, but they were within the physiological range. Low-titre viraemia was detected in 2/4 infected dogs between days 1 and 3 p.i. All infected dogs developed a robust immune response, in terms of neutralising antibodies. Thus, TBEV infections lead to effective seroconversion in dogs. Next, to assess TBEV exposure in dogs in the TBEV-endemic region of the Czech Republic, we conducted a serosurvey. Virus neutralisation tests revealed TBEV-specific antibodies in 17 of 130 (13.07%) healthy dogs, which confirmed a high, but clinically inappreciable TBEV exposure rate in the endemic area. The seropositivity rate was similar (12.7%; 41 positives out of 323) in a subgroup of dogs with various clinical disorders, and it was 13.4% (23 out of 171) in a subgroup of dogs with signs of acute neurological disease. Two dogs with fatal acute meningoencephalitis showed positive results for TBEV-specific IgM and IgG antibodies. These data extended our understanding of the clinical presentation of TBEV infections.

## 1. Introduction

Tick-borne encephalitis (TBE) is a zoonotic disease caused by the tick-borne encephalitis virus (TBEV; family *Flaviviridae*, genus *Flavivirus*) [1]. TBEV is spread primarily by tick bites. In nature, ticks, together with small rodents, serve as the main reservoirs of TBEV [2,3]. Although several tick species can serve as competent vectors of TBEV, *Ixodes ricinus* ticks are the primary TBEV vector in Europe. Ticks, including *I. ricinus*, are common ectoparasites of dogs, and consequently, dogs are frequently infected with TBEV [4]. However, dogs are only accidental hosts, and they cannot spread the virus further; thus, they do not play a role in the circulation of the virus in the natural setting. Serological studies in dogs have indicated that, frequently after a TBEV infection, seroconversion occurs without any clinical signs of TBE [5,6,7,8,9,10,11,12,13]. Based on those findings, dogs seem to be less susceptible to clinical TBE than humans [4,14].

To date, only a few cases of clinically manifest TBE have been reported in dogs. These manifestations typically take a monophasic course, after an incubation period ranging from 5–9 days [4,15,16]. In 16–50% of manifest TBE cases in dogs, the outcome is fatal [16]. Initially, the infected animals are depressed and show nonspecific signs that may include salivation, vomiting, aphagia, general weakness, unusual behaviour, including apathy or uncharacteristic or increased aggressiveness, and head pressing [9,17]. Soon after these nonspecific signs, neurological complications typically appear, and multifocal neurological impairments in the cerebrum and brain stem may develop. The disease is also characterised by elevated body temperature (up to 41.4 °C). This is initially due to fever, but later, it is more likely due to nonvoluntary excessive muscle contractions [9,17]. The reasons for these rare clinical presentations with frequent fatal outcomes remain unknown. It is likely that the dog’s breed, age, immune and general health status, the particular TBEV strain, and the infectious dose may play a role in determining the clinical course of the infection.

In this study, we aimed to determine the clinical signs of TBEV infections in dogs. We experimentally infected three-month-old beagle dogs with TBEV and measured body temperature, clinical signs, blood haematology, blood biochemistry, and immune responses. In addition, we conducted a serological survey to assess TBEV exposure in dogs in the Czech Republic.

## 2. Materials and Methods

### 2.1. Viruses and Cells

We experimentally infected dogs with the TBEV strain 9001 (isolated from a pool of *I. ricinus* ticks collected near Prague in 1978 in Czechoslovakia), which was passaged twice in the brains of suckling mice, then once in porcine stable kidney (PS) cells, and then once again in the brains of suckling mice. In the virus neutralisation test, we used the TBEV strain, Hypr (Czech prototype strain, originally isolated from the blood of a diseased 10-year-old child with TBE in 1953 in Czechoslovakia), which was passaged five times in the brains of suckling mice and once in PS cells. The viruses were provided by the Collection of Arboviruses, Biology Centre of the Czech Academy of Sciences.

PS cells [18] were cultured in Leibovitz L-15 Medium (Biosera, Nuaillé, France) supplemented with 3% foetal bovine serum (Gibco, Waltham, MA, USA), 100 U/mL penicillin, 100 µg/mL streptomycin, and 1% glutamine (Biosera, France).

### 2.2. Experimental Infection of Dogs

Six beagle dogs (females, three months old) were used in this study. The dogs were housed in an animal facility in a room that maintained a constant ambient temperature (15–24 °C) and a day/night cycle of 12/12 h. A standard feed mixture for the dogs was provided and drinking water was available ad libitum. Two dogs were subcutaneously inoculated with TBEV at a dose of 10^8^ PFU/per dog (high dose group); two dogs were subcutaneously inoculated with a dose of 10^6^ PFU/per dog (low dose group); and two dogs were subcutaneously inoculated with the vehicle (culture medium) only (control group). The dogs were inoculated behind the left shoulder blade. Clinical observations were evaluated daily during a four-week experimental period. The rectal temperature of the dogs was measured daily for 14 days p.i. Blood samples were collected daily on days 0–8, and then on days 10, 12, 14, 21, and 28 p.i. On day 28 p.i., the animals were euthanised, a necropsy was performed, and tissue samples were collected. Samples of liver, spleen, cerebral cortex, cerebellum, medulla, and cerebrum were examined in plaque assays to determine the presence of TBEV. Additionally, a histopathological examination was performed to evaluate the pathology. Briefly, tissue samples (1 cm^3^) were fixed in 10% paraformaldehyde before sectioning for haematoxylin and eosin staining. A board-certified veterinary pathologist examined the tissues as a commercial service at the State Veterinary Institute Olomouc, Czech Republic.

The study was conducted according to the amended Act No. 246/1996 on Animal Health and Animal Welfare of the Czech Republic. The use of animals in the experiment was approved by the Ministry of Agriculture of the Czech Republic (Approval No. 59811/2019-MZE–18134; date 17 January 2019).

### 2.3. Blood Haematology and Biochemistry

Blood samples were collected with anticoagulant (K3EDTA) and tested with an automated haematology analyser, Celltac Alpha (Nihon Kohden, Tokio, Japan). The following parameters were measured: white blood cell count, eosinophil count and percentage, red blood cell count, haemoglobin, haematocrit, mean corpuscular volume, mean corpuscular haemoglobin, mean corpuscular haemoglobin concentration, lymphocyte count and percentage, monocyte count and percentage, granulocyte count and percentage, red cell distribution width, procalcitonin test, mean platelet volume, platelet distribution width, and platelet count.

Sera prepared from the blood samples were analysed with an automated biochemistry analyser (Cobas 6000 C501; Roche, Basel, Switzerland). The following biochemical parameters were measured: total protein, albumin, gamma-glutamyl transferase, bilirubin, cholesterol, alanine aminotransferase, glucose, calcium, phosphorus, creatinine, and blood urea nitrogen.

### 2.4. Plaque Assay

The TBEV titres in sera of the infected dogs were determined in a plaque assay with PS cells, as described previously [19], with some modifications. Briefly, 2.1 mL of 10× diluted serum (in culture medium) was applied to monolayers of PS cells grown in 24-well tissue culture plates (30 µL sera + 270 µL culture medium per well; 7 wells per sample). Cells were incubated for 2 h at 37 °C. After the incubation, 200 µL of culture medium and 400 µL of 1.5% carboxymethylcellulose was added to each well. After a 5-day incubation at 37 °C, the cell monolayers were visualised with naphthalene black (0.1% naphthol blue-black (Sigma-Aldrich, Burlington, MA, USA), 6% glacial acetic acid (Sigma-Aldrich, Burlington, MA, USA) and 1.36% sodium acetate (Sigma-Aldrich, Burlington, MA, USA)). TBEV titres were expressed as the number of plaque-forming units (PFU/mL).

### 2.5. ELISA

Specific antiTBEV IgG antibodies in serum samples were detected with the IMMUNOZYM FSME IgG All Species kit (PROGEN GmbH, Heidelberg, Germany) according to manufacturer instructions. The antiTBEV IgG antibody concentrations are expressed in Vienna units (VIEU/mL). The detection of antiTBEV-specific IgG antibodies was considered negative for samples with less than 63 VIEU/mL; borderline positive for samples with titres of 63 to 126 VIEU/mL; and positive for samples with titres >126 VIEU/mL.

Specific dog antiTBEV IgM and IgG antibodies in serum samples were detected with the IMMUNOZYM FSME IgG All Species kit (PROGEN GmbH, Germany) with some modifications. Instead of the conjugate provided by the manufacturer, we used goat antidog IgM conjugated with horseradish peroxidase (IgM-HRP) or goat antidog IgG-HRP (diluted 1:10,000; Bethyl Laboratories). Specific dog antiTBEV IgM or IgG were detected with a spectrometer at 450 nm. Specific antiTBEV IgM or IgG antibody titres were defined as the serum dilution that gave an absorbance value twice that of the absorbance value for the same dilution of a negative control serum.

### 2.6. Virus Neutralisation Test

Specific antiTBEV-neutralising antibodies were measured in serum samples with the virus neutralisation test (VNT), as described previously [20]. Briefly, sera were diluted 1:4 in an L-15 Medium (Biosera, Nuaillé, France) supplemented with 3% foetal bovine serum (Gibco, USA), 100 U/mL penicillin, 100 µg/mL streptomycin, and 1% glutamine (Biosera, Nuaillé, France). Then, sera were inactivated for 30 min at 56 °C. Next, 2-fold serial dilutions of the sera in an L-15 Medium (50 µL/well) were incubated with 10^3^ PFU/mL of the Hypr TBEV strain (50 µL/well) in 96-well plates (TPP, Trasadingen, Switzerland) for 90 min at 37 °C. The dose of TBEV was adjusted to cause a cytopathic effect, with near complete (90–95%) cytolysis. Next, PS cells were added (3 × 10^4^ cells in 100 µL/well). After a 5-day incubation at 37 °C, the cytopathic effect was investigated with an inverted microscope (Olympus). Serum samples with titres ≥1:20 were considered positive.

### 2.7. Collection of Dog Serum for Serological Surveillance

Serum samples were collected for serological surveillance from 130 healthy dogs and 323 dogs with diverse health impairments that were treated at the University of Veterinary Sciences, Brno (Czech Republic) from 2016–2020. Samples were collected as part of a routine or specialised veterinary investigation. In addition, cerebrospinal fluid (CSF) was collected from two dogs with acute meningoencephalitis. Sera were stored at −80 °C before analysis. Sera were tested for specific antiTBEV antibodies with an ELISA, as described above (Section 2.5). All samples were subsequently analysed with a VNT, as described above (Section 2.6).

### 2.8. Statistical Analyses

Differences in VNT seroprevalence between groups were tested with generalised linear models (GLZ) with a binomial distribution. The effect of age on VNT seroprevalence was tested with the Spearman correlation test for arcsine-transformed prevalence data (age groups that contained at least 10 dogs/group were included in the analysis). Data were analysed with Statistica for Windows, version 13.0. *p* values < 0.05 were considered statistically significant.

## 3. Results

### 3.1. Experimental Infection of Dogs

**Figure 1:**
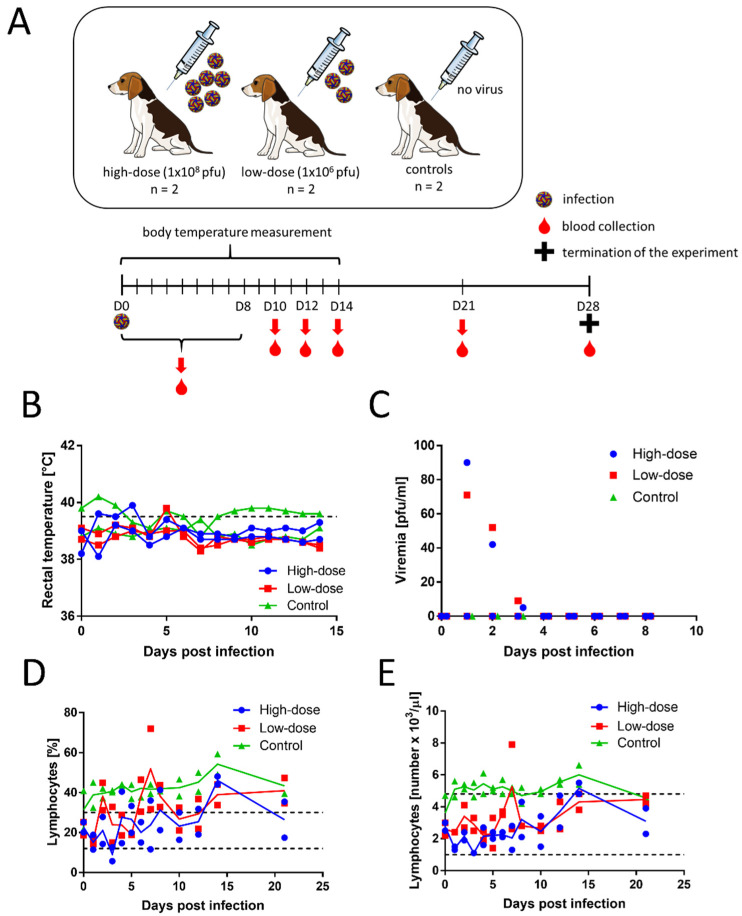
Experimental TBEV infections in dogs. (**A**) Experimental protocol: Six beagle dogs (females, three months old) were included. Dogs were subcutaneously inoculated with TBEV at 10^8^ PFU/per dog (high-dose group, *n* = 2, *blue circles*), TBEV at 10^6^ PFU/per dog (low-dose group, *n* = 2, *red squares*), and no TBEV (control, *n* = 2, *green triangles*). Blood samples were collected daily on days 0–8, then on days 10, 12, 14, 21, and 28 p.i. Animals were euthanised at 28 p.i. (Figure created with Servier Medical Art, available at www.servier.com). (**B**) Individual daily rectal temperatures are shown for 14 days p.i. The dashed line indicates the threshold for fever (39.5 °C). (**C**) Individual TBEV titres measured on the indicated days, determined with plaque assays. The limit of detection was 4.7 pfu/mL. (**D**,**E**) Individual blood lymphocyte levels, measured with an automated haematology analyser on the indicated days, show (**D**) lymphocyte percentages and (**E**) lymphocyte counts. Dashed lines indicate the upper and lower limits for the healthy condition.

Four female beagle dogs (3 months old) were subcutaneously inoculated with TBEV, strain 9001. Two female dogs of the same breed and age were used as controls (Figure 1A). After the inoculations, none of the infected dogs showed any clinical signs of the disease. Rectal temperature was measured daily for 14 days p.i. The temperatures ranged from 38.1 to 39.9 °C in the inoculated dogs and 38.7 to 40.2 °C in control dogs (Figure 1B). For dogs, fever is defined as a temperature above 39.5 °C. The slightly elevated rectal temperatures were likely caused when the measurement was taken after the animals had been actively running around the room.

Blood samples were collected daily on days 0–8, and then on days 10, 12, 14, 21, and 28 p.i. Serum viraemia was detected with plaque assays in 2 dogs (one in the low-dose group and one in the high-dose group) on days 1–3 (Figure 1C). In both dogs, the virus titres were low, and the peak titres were measured on day 1 (1.85 and 1.95 log_10_ PFU/mL). No virus was isolated in the plaque assay from the sera of the other animals.

Results from the haematological and biochemical analyses are shown in Supplementary Materials. We found no significant changes in most measured haematological and biochemical parameters, and most measurements remained within the physiological range. The infected dogs had only slightly lower blood lymphocyte counts than the controls (Figure 1D,E), but the values were within the physiological range. All animals had high platelet levels, even before virus inoculation, and the levels remained high during the entire experiment. The albumin levels were also above physiological range in all analysed animals, including controls. Some of the infected animals showed cholesterol and glucose levels that were slightly increased over the physiological range. However, the albumin, cholesterol, and glucose levels could have been affected by diet, rather than by the infection. The haematology and blood biochemistry parameters were not substantially different between the low- and high-dose groups.

**Figure 2:**
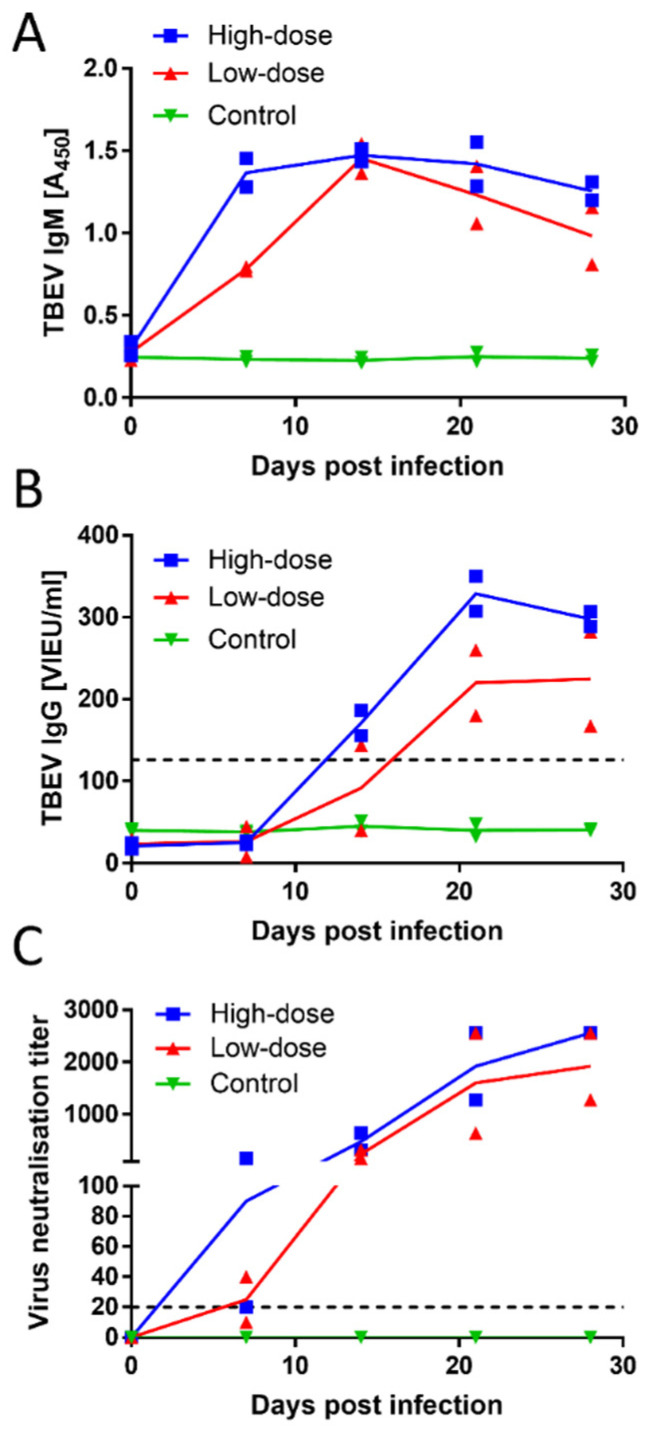
Antibody responses in dogs experimentally infected with TBEV. Six beagle dogs were included. TBEV was inoculated at 10^8^ PFU/per dog (high-dose group, *n* = 2, *blue squares*), TBEV at 10^6^ PFU/per dog (low-dose group, *n* = 2, *red triangles*), or no TBEV (control group, *n* = 2, *green inverted triangles*). Blood samples were collected on days 0, 7, 14, 21, and 28 p.i. ELISA results show (**A**) TBEV-specific IgM, and (**B**) IgG levels in blood sera from individual dogs; (**C**) Individual levels of TBEV-neutralising antibodies, assayed with VNTs. The dashed lines in (**B**,**C**) indicate thresholds for positive antibody detection.

All animals were seronegative for TBEV at the time of inoculation, as demonstrated by both ELISA and VNT. Dogs in both experimental groups (low- and high-dose) developed a robust antibody response, based on ELISA and VNT measurements. The inoculated dogs showed positive results for TBEV IgM as early as day 7 p.i., and they remained positive over the entire experimental period (Figure 2A). Three out of the four inoculated animals showed positive results for TBEV IgG antibodies on day 14 p.i. On day 21 p.i., all four inoculated animals were positive for the presence of TBEV IgG antibodies (Figure 2B). Neutralising titres in three of the four infected dogs reached or exceeded 1:20 as early as day 7 p.i. All four inoculated animals were positive for virus-neutralising antibodies on day 14 p.i. The titres increased with time, reaching or exceeding titres of 1:1280 at the time of experiment termination on day 28 p.i. (Figure 2C). No substantial differences in antibody titres were observed between dogs given a low or high dose of the virus. The control group showed negative results in the ELISA and the VNT throughout the entire experiment.

At necropsy, on day 28 p.i., samples from the liver, spleen, cerebral cortex, cerebellum, cerebrum, and medulla were collected and investigated by a board-certified veterinary pathologist. No significant pathological changes were found in any analysed samples, from either inoculated or control animals.

### 3.2. Seroprevalence Study

**Figure 3:**
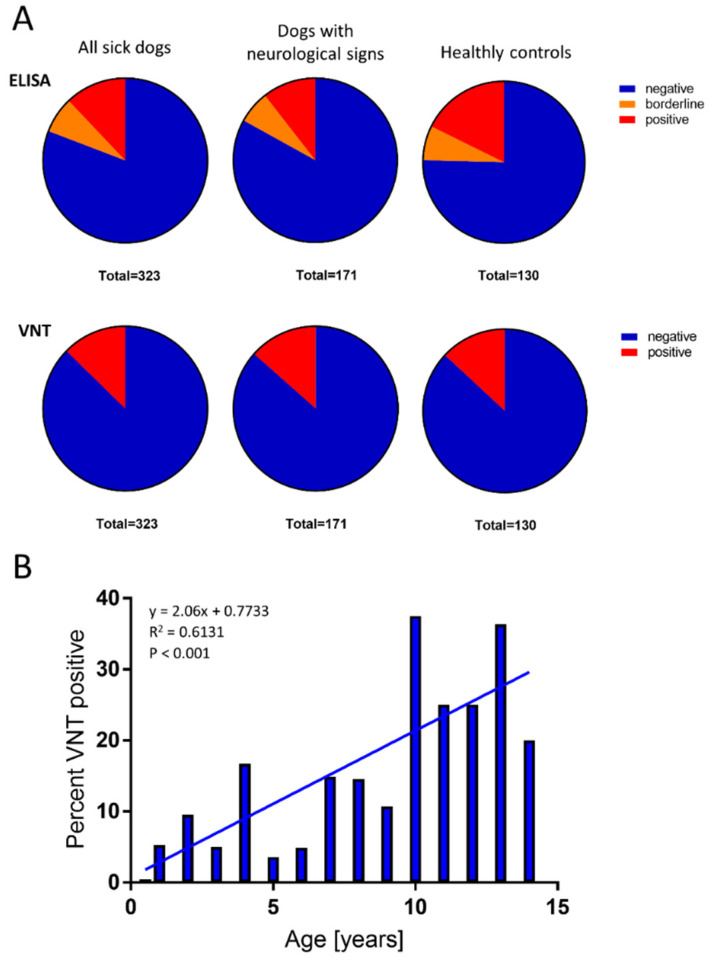
TBEV seroprevalence study in dogs. Serum samples were collected from 453 dogs in the Czech Republic. (**A**) Pie charts show the results of TBEV tests among healthy dogs (*n* = 130, *right*) and dogs investigated for various health problems (all sick dogs, *n* = 323, *left*); among the latter, 171 had signs of neurological impairment (dogs with neurological signs, *middle*). Each pie chart shows the proportion of dogs in each group with TBEV-specific IgG antibodies (ELISA results, *upper row*) and virus-neutralising antibodies (VNT results, *lower row*). (**B**) Correlation analysis shows the relationship between TBEV seropositivity rates and dog age (correlation curve is shown). Age groups comprised at least 10 dogs each.

A total of 453 sera were collected from dogs in the Czech Republic, which is endemic for TBE. The sera were collected from either healthy dogs (*n* = 130) or dogs that had been investigated for various health problems (*n* = 323; of these, 171 dogs had signs of neurological impairment). Out of the 130 healthy dogs, 23 (17.69%) showed positive ELISA results for TBEV IgG, and of these, 17 (13.07%) showed positive VNT results for TBEV-neutralising antibodies. A similar level of seroprevalence was observed in dogs with health impairments (*n* = 323); 39 (12.07%) showed positive ELISA results for TBEV IgG, and 41 (12.7%) showed positive VNT results for TBEV-neutralising antibodies. Among the 171 dogs (10.53%) with neurological impairments, 18 showed positive ELISA results for TBEV IgG, and 23 (13.4%) showed positive VNT results (Figure 3A). No statistically significant differences in the seroprevalence of virus-neutralising antibodies were found between the groups investigated (*p* > 0.05).

The ages of all 453 dogs ranged from 0.5 to 15 years (average 6.0 years). The average age of dogs with positive VNT results was 8.0 years. We identified a significant correlation (*p* < 0.001) between age and VNT positivity (Figure 3B). No statistical difference in TBEV seroprevalence was found between males (13.7%) and females (11.8%).

### 3.3. Clinical Presentation of Natural TBEV Infections in Dogs

We retrospectively tested serum samples collected from two male dogs with acute meningoencephalitis (Rottweiler, five years old; German shepherd, six years old). Both dogs were positive for antiTBEV IgG and IgM antibodies, based on ELISAs. In addition, the Rottweiler was positive for antiTBEV IgG in the CSF. In both dogs, antiTBEV IgM was detected in the CSF.

The Rottweiler could not stand, and was lying only in the lateral position with progressive disorientation, polypnoea, bronchial breathing, and variable positional strabismus. His body temperature was 43.0 °C. He was hospitalised for two days at the University Veterinary Hospital. During hospitalisation, he showed signs of progressive stupor, excitation, polypnoea, and dyspnoea, which ultimately led to a fatal outcome. Neurological signs indicated multifocal damage in the brainstem and cortex. A haematology analysis revealed leucocytosis/neutrophilia with a left shift. The blood biochemistry indicated normal values, except for elevated C-reactive protein (CRP) levels (70.48 mg/L; normal values ≤5 mg/L). A CSF analysis revealed inflammatory signs, with activated lymphocytes and monocytes, and markedly high levels of total protein (2.664 g/L; normal values ≤0.20–0.45 g/L) and albumin (0.453 mg/L; normal values ≤0.120–0.300 mg/L). In addition, the CSF had low glucose levels (1.2 mmol/L). ELISAs revealed TBEV-specific IgG (140 VIEU/mL, titre 1:128,000) and IgM (titre 1:256,000) antibodies in the serum and antiTBEV IgG (155 VIEU/mL, titre 1:64,000) and IgM (titre 1:16,000) antibodies in the CSF. A VNT confirmed the presence of antiTBEV antibodies in the serum, with a titre of 1:1280.

The German shepherd was admitted to the University Veterinary Hospital after two days of lethargy, vomiting, weakness in the hind limbs, inability to stand or walk, and fatigue. At the time of hospitalisation, the dog showed signs of polypnoea or dyspnoea, thoracic limbs myoclonus, and later, myoclonus in the pelvic limbs and generalised seizures. The dog was hospitalised for three days, and then was terminated by euthanasia. During hospitalisation, the dog’s body temperature was 39.5–41.2 °C, and he presented with hyperaemia in the mucous membranes and conjunctiva, myoclonus, and increased muscle tone. A neurological investigation revealed an excitation, stupor, mimic myoclonus, aggression, and biting. The dog was lying in the lateral recumbent position. He showed signs of spontaneous nystagmus. A haematology examination revealed lymphocytopaenia. The blood biochemistry was normal, except for elevated CRP levels (78.8 mg/L; normal value ≤5 mg/L). A serological investigation showed negative results for anti*Neospora* IgM and IgG antibodies, anti*Toxoplasma* IgM antibodies, and anti*Anaplasma* IgM antibodies. However, they found borderline detection of anti*Toxoplasma* IgG antibodies and anti*Borrelia* IgM and IgG antibodies, and positive detection of anti*Anaplasma* IgG antibodies. A CSF analysis revealed inflammatory signs, with activated lymphocytes and monocytes, and increased levels of total protein (1.7 g/L; normal values ≤0.20–0.45 g/L) and albumin (0.354 mg/L; normal values ≤0.120–0.300 mg/L). A TBEV ELISA indicated borderline positive results for antiTBEV IgG antibodies in the serum (86 VIEU/mL; titre 1:64,000) and positive results for antiTBEV IgM antibodies (titre 1:64,000). An ELISA of CSF also showed specific antiTBEV IgM antibodies (titre 1:16,000) and low levels of antiTBEV IgG antibodies (49 VIEU/mL, titre 1:8000). A VNT confirmed the presence of antiTBEV antibodies in the serum, with a titre of 1:1280.

## 4. Discussion

TBE is known to cause severe or even fatal encephalitis in dogs. Ticks are frequent parasites of dogs; their furry coats efficiently collect ticks from grass and undergrowth. It is estimated that the probability of coming into contact with a tick is about 50–100-fold higher in dogs than in humans [4]. However, it seems that dogs are less susceptible than humans to clinically manifest TBE, because only a few reports on clinical TBE in dogs have been reported to date [4,9,15,16]. Of note, the cases of clinical TBE in dogs are typically severe, and the outcome is frequently fatal. Dogs with a peracute course often die within three to seven days [15]. It remains unknown why most infected dogs develop no disease, but sporadic cases are severe or lethal.

Here, we addressed this issue by experimentally infecting dogs with TBEV. Although two of four infected dogs exhibited detectable low-titre viraemia, we found no significant clinical signs or pathological features associated with the infection. Infected dogs showed no clinical signs, and most haematology and blood biochemistry parameters were unaffected by the infection. The only exception was a slightly reduced blood lymphocyte count in the infected animals, which might reflect the infection response observed in humans, where leucocytopaenia frequently occurs during the first (viraemic) phase of TBE [21]. However, all infected dogs developed a robust immune response in terms of neutralising antibodies. These results were in good agreement with previous reports on experimental infections in dogs published in the 1970s [22,23]. It is also of interest that only 3 out 4 infected dogs had measurable TBEV-specific IgG antibodies in their sera on day 14 p.i., but all sera efficiently neutralised the virus. This indicates that IgM antibodies can contribute to sufficient neutralisation of TBEV.

Two previous studies experimentally infected dogs with TBEV. In the first study, six to eight week-old puppies were infected with TBEV, either by subcutaneous inoculation or by allowing TBEV-positive ticks to feed on the dogs. In that study, three of the infected animals showed no clinical signs of the disease, but one puppy showed weakness in the extremities. No rise in temperature was observed. Low-titre viraemia was detected, irregularly, from day 1 to day 7 p.i. At autopsy, no virus was isolated from either the CNS or visceral organs, and no histopathological changes typical of TBE were observed. Nevertheless, seroconversion was observed in all animals, starting from the first week after infection [22]. In the other study, three puppies were inoculated subcutaneously with a high dose of TBEV. Again, no clinical signs were observed, and no histological changes were detected in the liver, spleen, kidney, or brain. No signs of persistent viral replication were observed, but all animals exhibited seroconversion [23].

One potential limitation of our study was that the dogs were infected with a subcutaneous inoculation of the virus in suspension, rather than allowing infected ticks to feed on the animals. Tick saliva contains a plethora of pharmacologically active compounds that modulate the microenvironment at the site of tick feeding. These compounds may enhance the infectivity of the tick-borne pathogens, including TBEV [24,25]. Furthermore, intradermal inoculation of the virus could be closer to the natural way of infection by ticks. However, in the experiments performed by Gresikova et al. [22], no significant differences were observed between dogs infected with TBEV by subcutaneous inoculation and dogs infected by allowing TBEV-positive ticks to feed on the dogs.

Our study results were consistent with those from studies conducted in the 1970s, which indicated that young dogs infected with TBEV showed asymptomatic disease, characterised by low-titre viraemia and a robust immune response, with no extensive virus replication in the periphery or CNS. Most clinically manifest TBE cases have been found in older dogs, and primarily in larger breeds [4,16,26,27]. However, it remains unknown whether age is the sole determinant of severity or whether some breeds are more sensitive than others. Moreover, the clinical course is likely to be affected by the dog’s overall health status, particularly the immune status and presence of co-morbidities.

Our second aim was to determine the seroprevalence of TBEV in a large cohort of dogs from the Czech Republic, which is known to be highly endemic for TBEV. Dogs were tested with both ELISAs and VNTs, because the TBE ELISA cannot fully replace VNT, particularly for epidemiological purposes [13]. We found similar levels of VNT seropositivity in healthy dogs (13.07%) and dogs with various health problems (12.7%). Importantly, the seroprevalence rate was similar (13.4%) among a subgroup of dogs with signs of acute neurological disorders. These results were not consistent with previous data from Germany, where TBE seroprevalence was substantially higher in dogs with neurological symptoms than in healthy dogs [5].

Previous serological surveys performed in dogs of various European countries revealed substantial differences in the prevalence of TBEV-specific antibodies found in different regions. However, the differences could be due to different methods used for the analyses. For example, the prevalence of TBEV infections among dogs determined with ELISAs were 31.5% in Germany [5], 11.3% in the Czech Republic [6], and 16.4% in Norway [7]. In Finland, the prevalence of TBEV infections among dogs was 6–40%, based on anti-TBEV IgG monoclonal antibody-capture and IgG immunofluorescence assays [8]. In the Czech Republic, the prevalence was 3.3%, based on a haemagglutination inhibition assay [9]. In contrast, prevalences determined with VNTs were 0.1% in Belgium [10], 4.8% in Denmark [11], 1.7% in Spain [12], and 22.1% in Germany [13]. The different prevalences of TBEV-infected ticks in different regions are thought to reflect the risk of TBEV infections. However, in addition to different detection methods, the results might also be affected by inclusion criteria and dog activity. For example, significantly higher seropositivity was found in hunting dogs than in pet dogs in Spain [12].

Our study showed that the TBEV seropositivity rates in dogs increased significantly with age. The average age of infected dogs (based on VNTs) was 8.0 years, consistent with findings from other studies performed with ELISAs in Norway (8.02 years) [7] and Denmark (8.1 years) [11]. This association could indicate two possibilities. Either antiTBEV antibodies are long lived after exposure; and thus, antibodies accumulated to higher levels in older animals, or exposure over an extended time increased the probability of TBEV-infected tick infestations [4].

Our retrospective examination of the epidemiological survey identified two dogs with acute TBE. Both dogs presented with a severe course of the infection, which was fatal (one experienced a natural death and the other received euthanasia). This finding was consistent with the notion that the development of severe TBEV symptoms almost exclusively results in a fatal outcome [4]. The dogs were 5 and 6 years old, consistent with previous case reports on TBE in dogs, which suggested that, empirically, older dogs seemed to be more sensitive to clinically manifest TBE [4,26]. Our CSF analyses in both dogs revealed inflammatory signs that are typical indicators of encephalitis and are commonly described in dogs with TBE [4,9]. The diagnoses were based on the simultaneous detection of TBEV-specific IgM and IgG antibodies in serum samples. The simultaneous appearance of antiTBEV IgM and IgG is typically used to diagnose TBE in humans [28]. In addition, antiTBEV IgM antibodies were detected in the CSF of both dogs, and antiTBEV IgG in one dog. Generally, CSF IgG antibodies are more specific than serum antibodies for diagnosing TBE in dogs [5,29].

As in human TBE cases, there is no specific antiviral drug available for treating TBE in dogs; thus, treatment aims to ameliorate symptoms. Consequently, preventive measures are highly important, and they are primarily based on preventing tick infestations in dogs [4]. Several highly effective antiectoparasite drugs containing acaricides are available on the market, but they fall short of providing complete protection. There is no TBEV vaccine licenced for animal use, including dogs. However, a candidate TBEV vaccine for veterinary use was recently developed, primarily for vaccinating goats and sheep [30]. If this vaccine becomes licenced, it could potentially be used for preventing TBEV infections in dogs.

## Data Availability

The data that supports the findings of this study are available from the corresponding author, J.S. or D.R., upon reasonable request.

## Supplementary Materials

Results from the haematological and biochemical analyses are available online at https://www.mdpi.com/article/10.3390/v13102039/s1.
