# Supplementary material for: Experimental and Natural Infections of Tick-Borne Encephalitis Virus in Dogs
Source: Viruses. 2021 Oct 9;13(10):2039. doi: 10.3390/v13102039 (PMC8537875; doi:10.3390/v13102039)
Supplement: Supplementary file 1 [file viruses-13-02039-s001.zip › viruses-1396457-supplementary.pdf]

Supplementary information

Dogs were subcutaneously inoculated with TBEV at 10^8 PFU/per dog (high-dose group, n=2, *blue circles*), TBEV at 10^6 PFU/per dog (low-dose group, n=2, *red squares*), and no TBEV (control, n=2, *green triangles*). Blood samples were collected daily on days 0-8, then on days 10, 12, 14, 21, and 28 p.i. Animals were euthanised at 28 p.i. Blood samples were tested with an automated haematology analyser, Celltac Alfa (Nihon Kohden). Sera prepared from the blood samples were analysed with an automated biochemistry analyser (Cobas 6000 C501; Roche).

WBC, white blood cells; **EO**, eosinophils; **RBC**, red blood cell count; **HGB**, haemoglobin; **HCT**; haematocrit; **MCV**, mean corpuscular volume; **MCH**, mean corpuscular haemoglobin; **MCHC**, mean corpuscular haemoglobin concentration; **LY**, lymphocytes; **MO**, monocytes; **GR**, granulocytes; **RDW**, red cell distribution width; **PCT**, procalcitonin test; **MPV**, mean platelet volume; **PDW**, platelet distribution width; **PLT**, platelets; **ALT**, alanine aminotransferase.

Levels of bilirubin and gamma-glutamyl transferase in all dogs investigated (infected or controls) were under the detection limit of the assays.

Dashed lines indicate the upper and lower limits for the healthy condition.

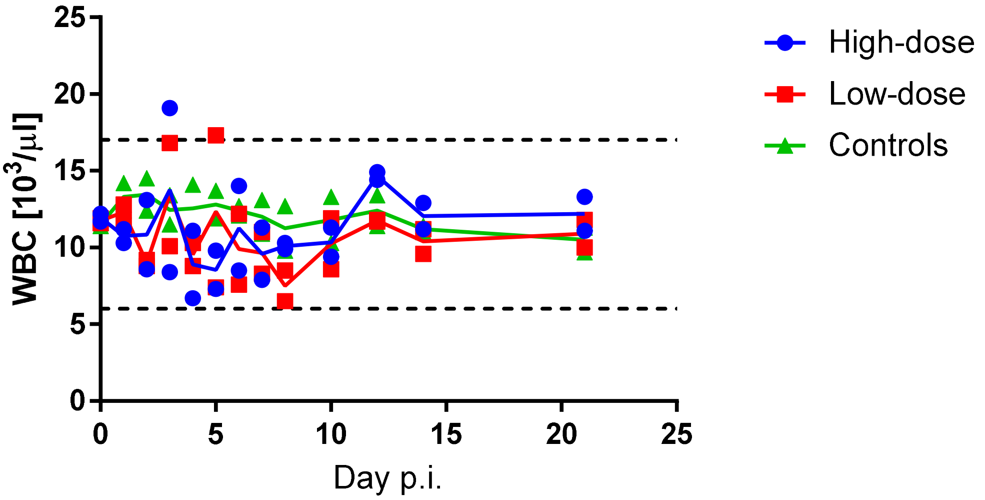

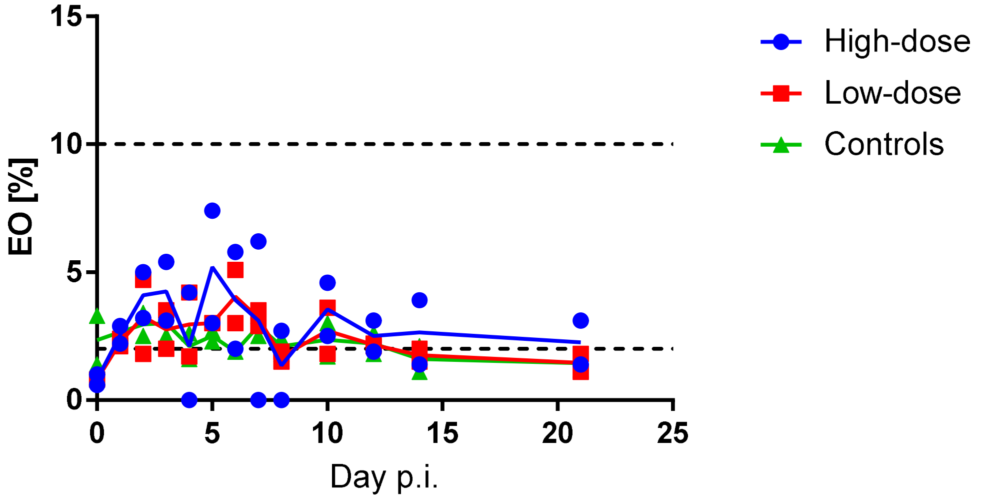

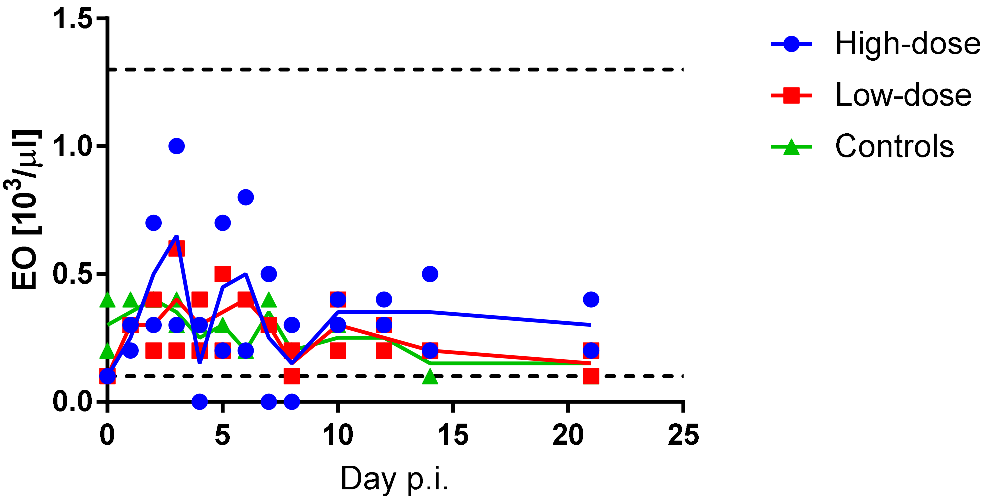
EO [10^3/µl]
1.5
1.0
0.5
0.0
0
5
10
15
20
25
Day p.i.
High-dose
Low-dose
Controls

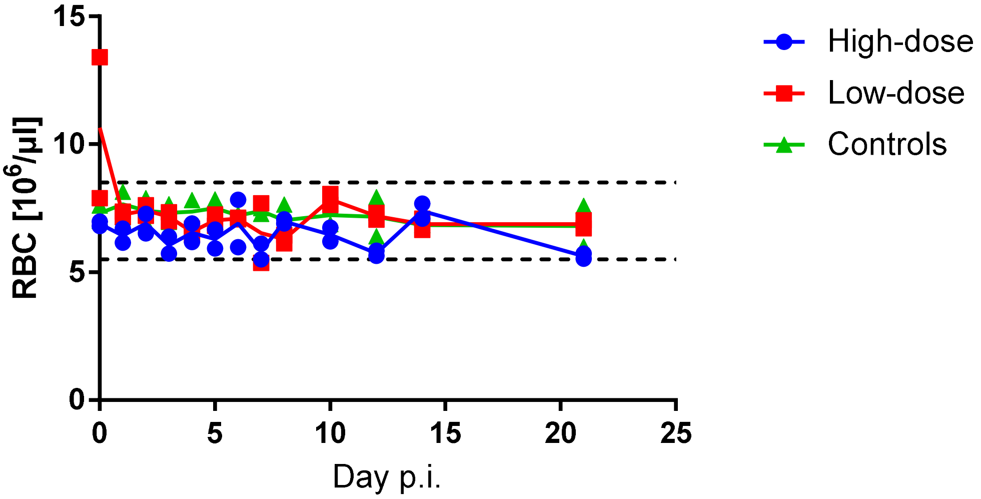
RBC [10^6/µl]
15
10
5
0
0
5
10
15
20
25
Day p.i.
High-dose
Low-dose
Controls

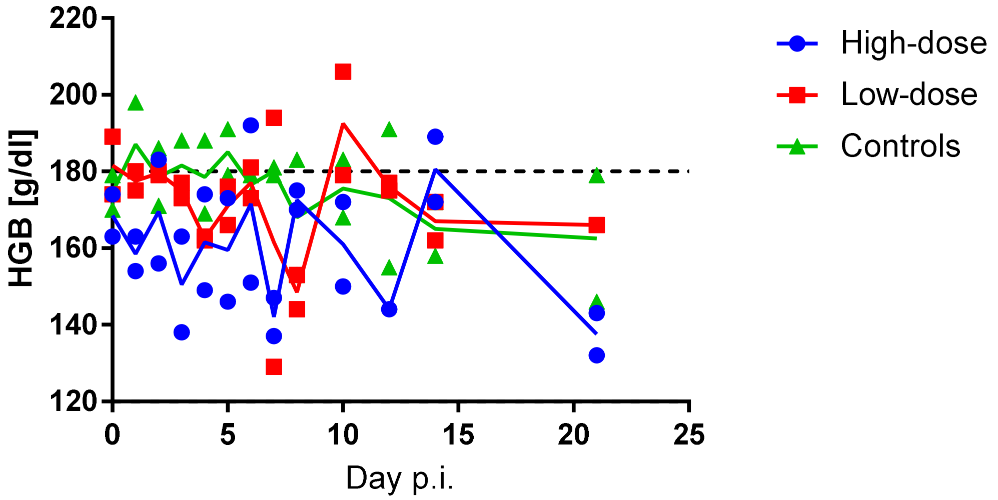
HGB [g/dl]
220
200
180
160
140
120
0
5
10
15
20
25
Day p.i.
High-dose
Low-dose
Controls

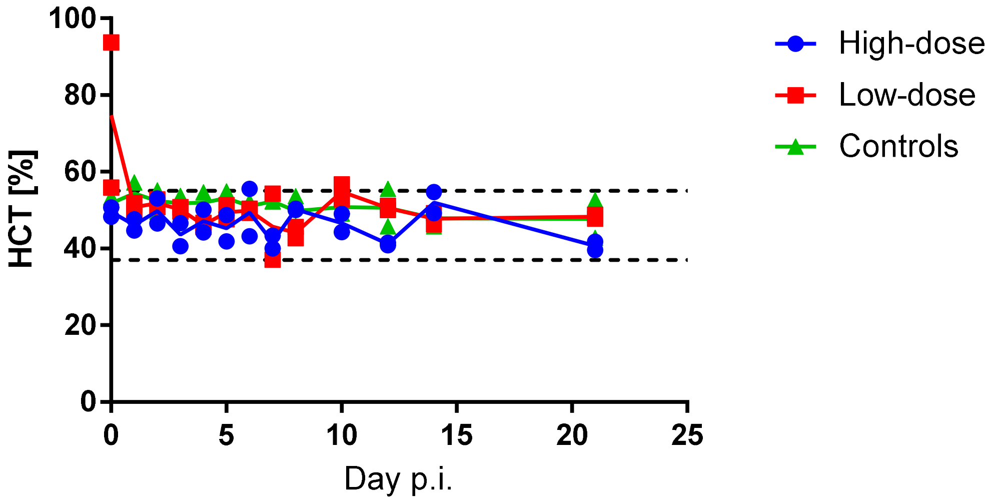
HCT [%]
Day p.i.
High-dose
Low-dose
Controls

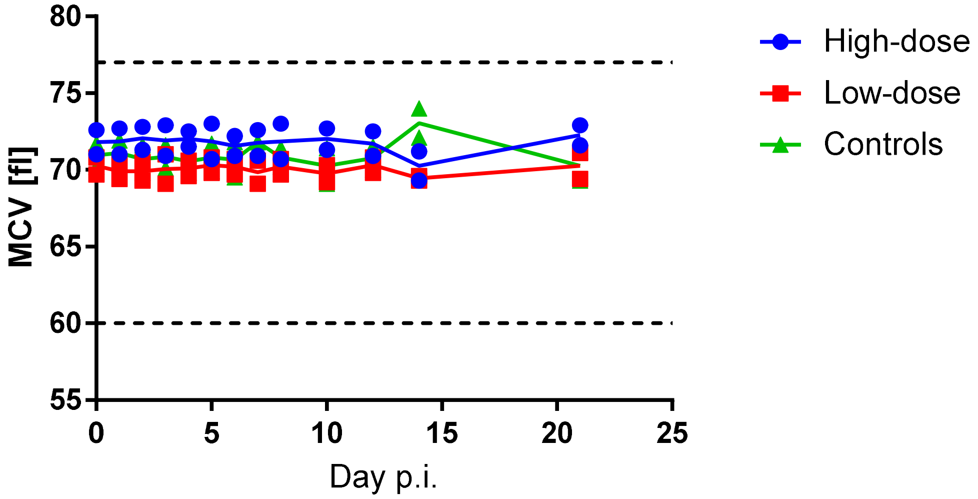
MCV [fl]
Day p.i.
High-dose
Low-dose
Controls

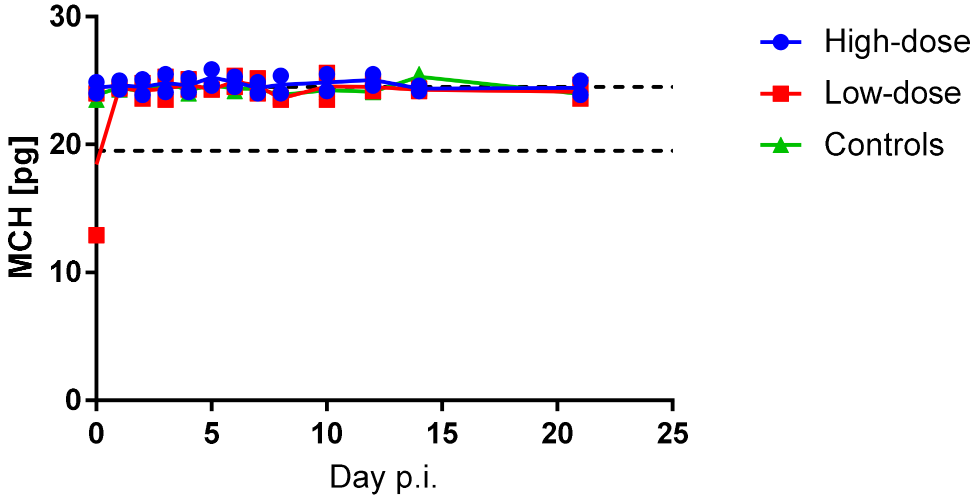
MCH [pg]
Day p.i.
High-dose
Low-dose
Controls

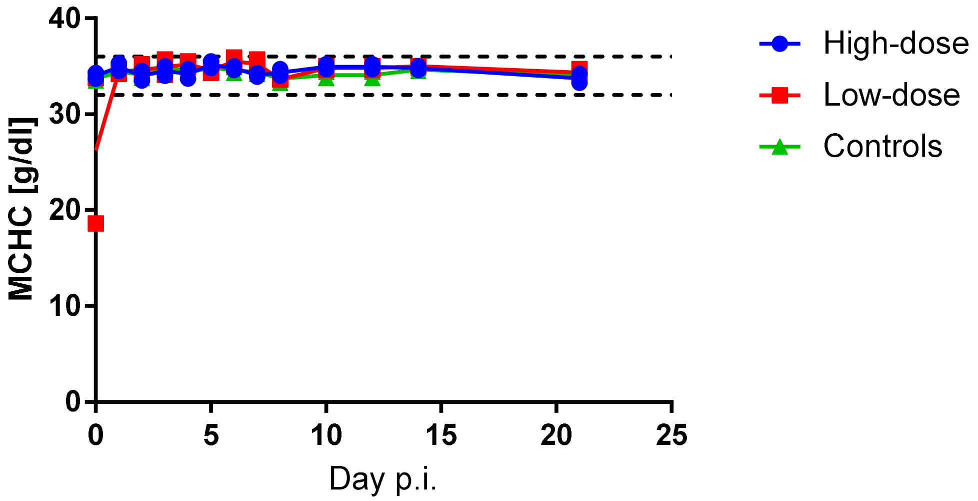

MCHC [g/dl]
40
30
20
10
0
0
5
10
15
20
25
Day p.i.
High-dose
Low-dose
Controls

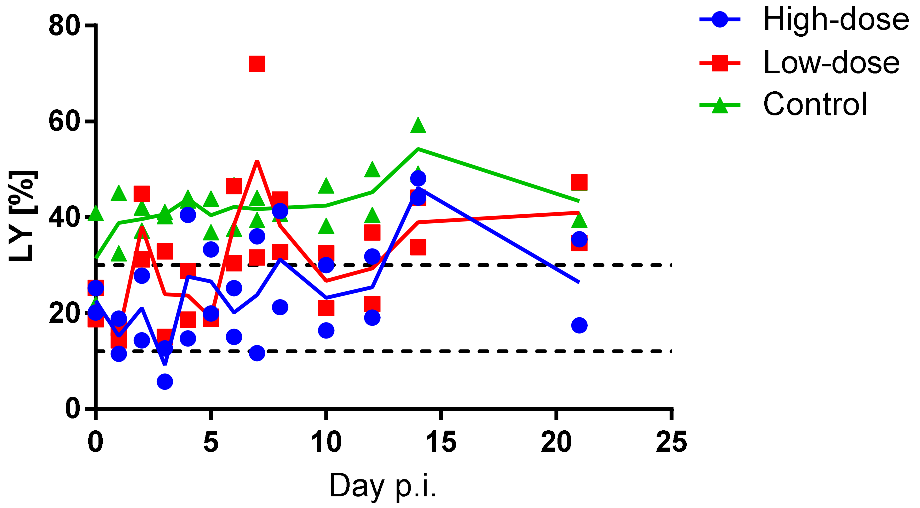

LY [%]
80
60
40
20
0
0
5
10
15
20
25
Day p.i.
High-dose
Low-dose
Control

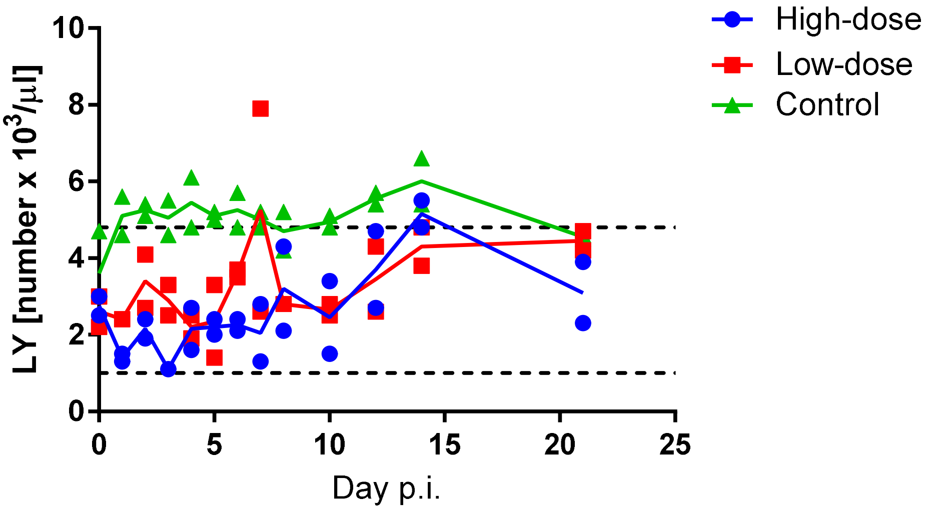

LY [number x 10^3/µl]
10
8
6
4
2
0
0
5
10
15
20
25
Day p.i.
High-dose
Low-dose
Control

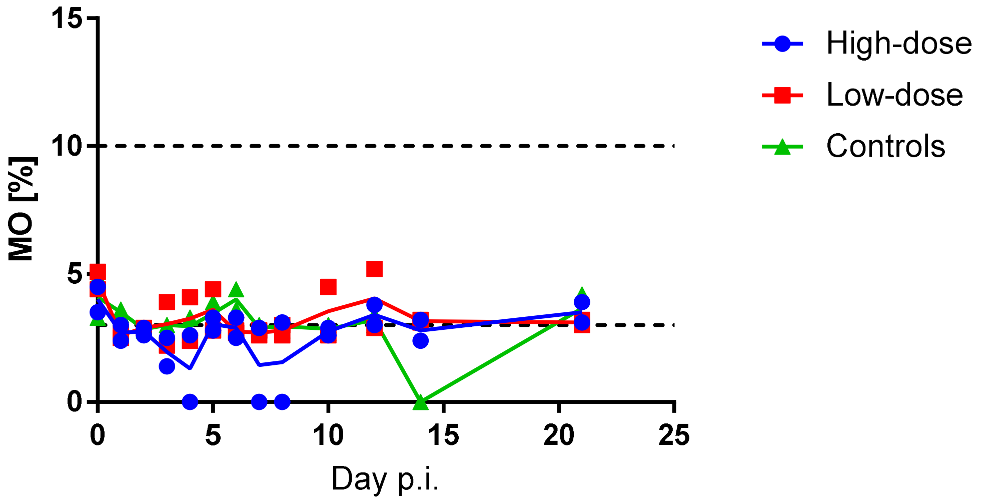

MO [%]
Day p.i.
High-dose
Low-dose
Controls

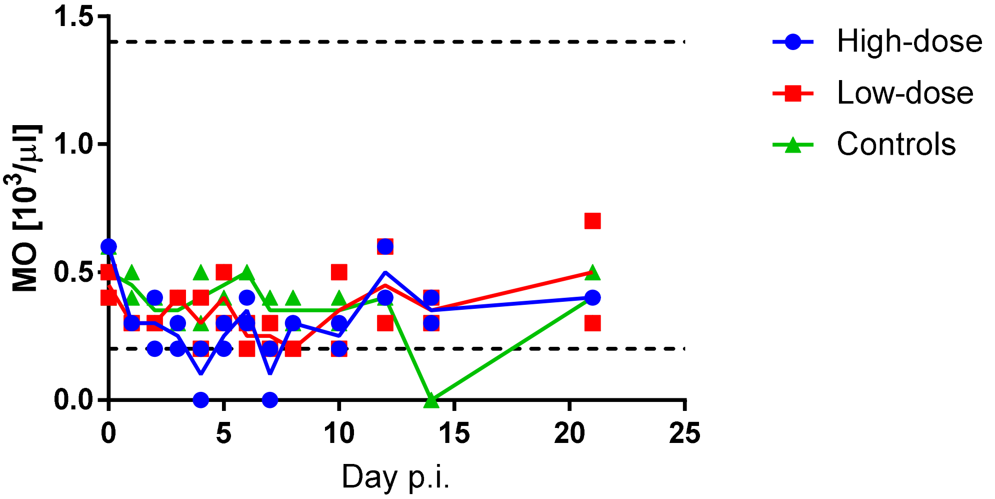

MO [10³/µl]
Day p.i.
High-dose
Low-dose
Controls

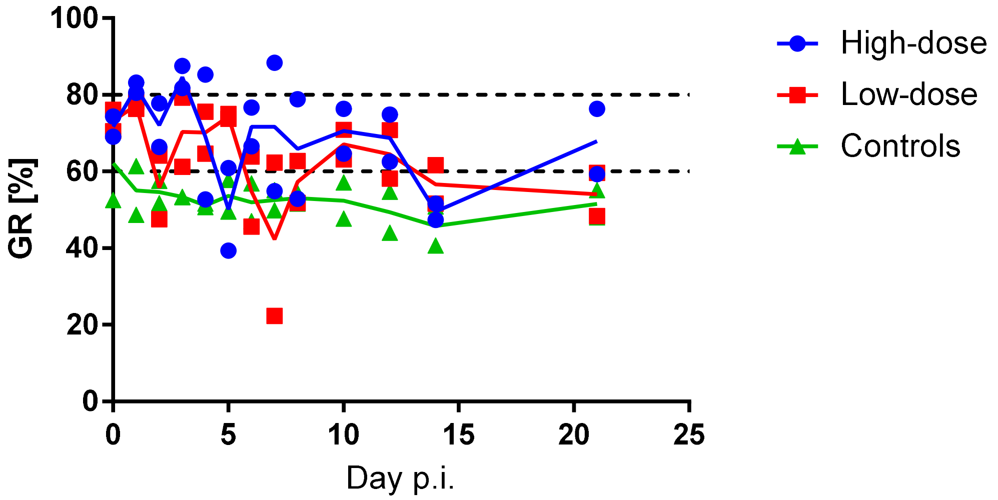

GR [%]
Day p.i.
High-dose
Low-dose
Controls

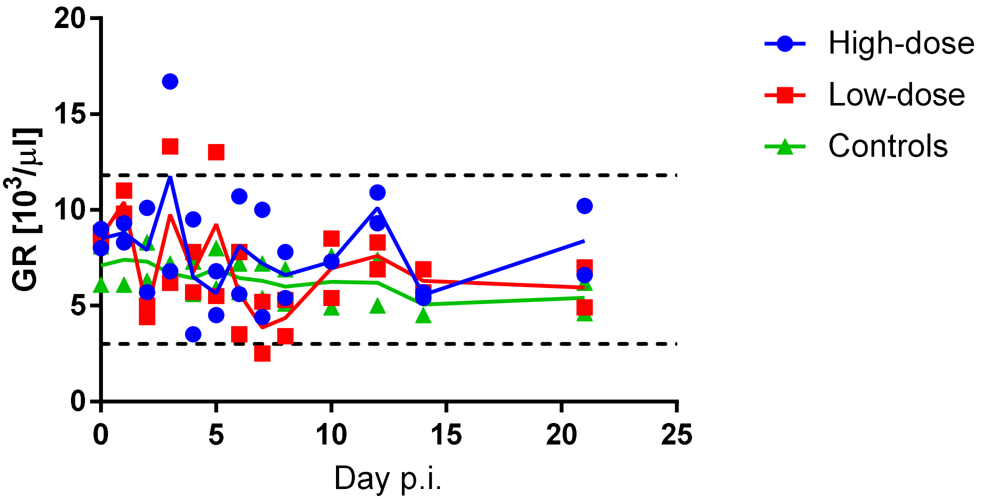

GR [10³/µl]
Day p.i.
High-dose
Low-dose
Controls

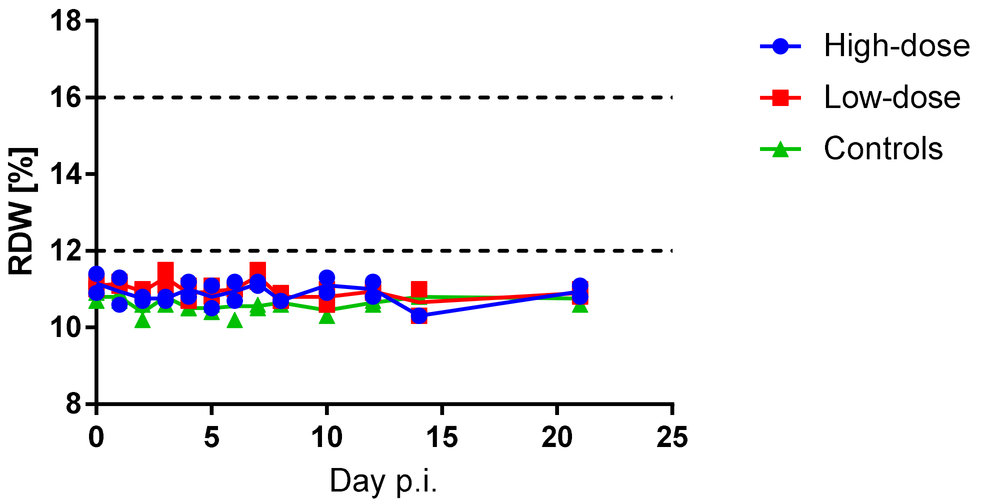

RDW [%]
Day p.i.
High-dose
Low-dose
Controls

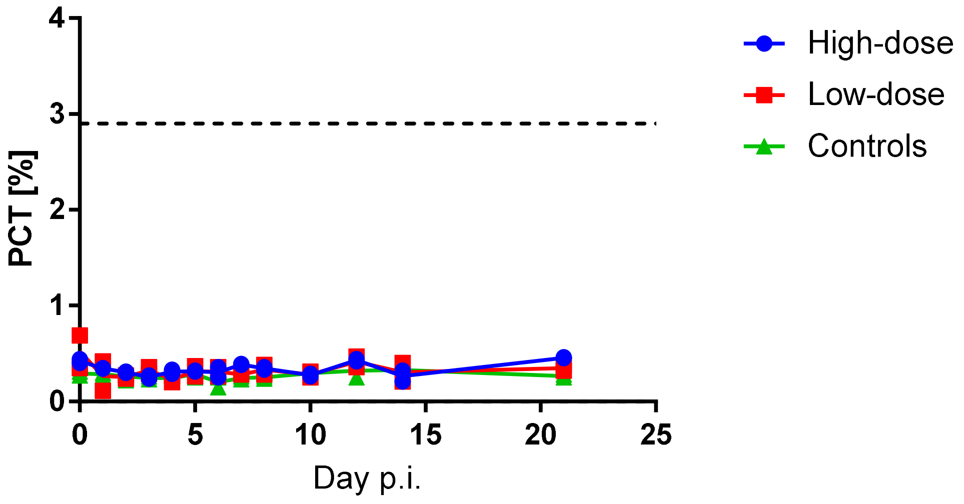

PCT [%]
Day p.i.
High-dose
Low-dose
Controls

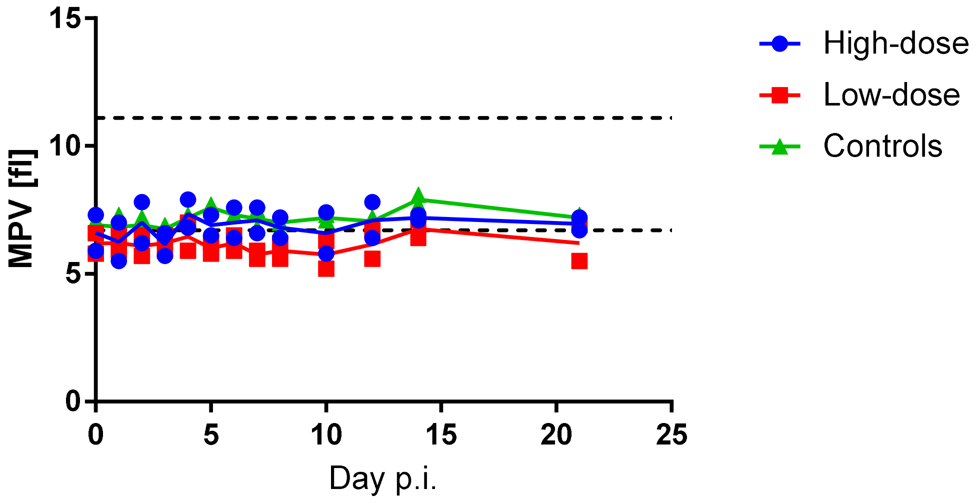

MPV [fl]
15
10
5
0
0
5
10
15
20
25
Day p.i.
High-dose
Low-dose
Controls

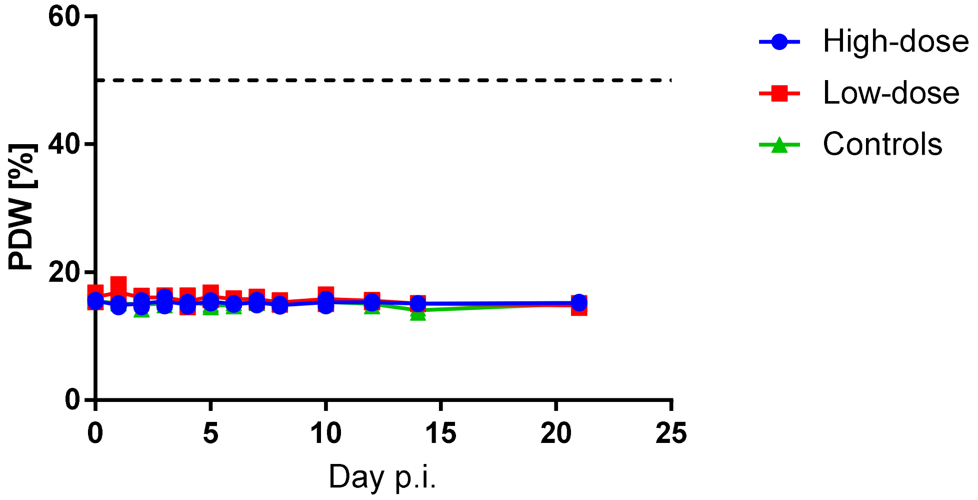

PDW [%]
60
40
20
0
0
5
10
15
20
25
Day p.i.
High-dose
Low-dose
Controls

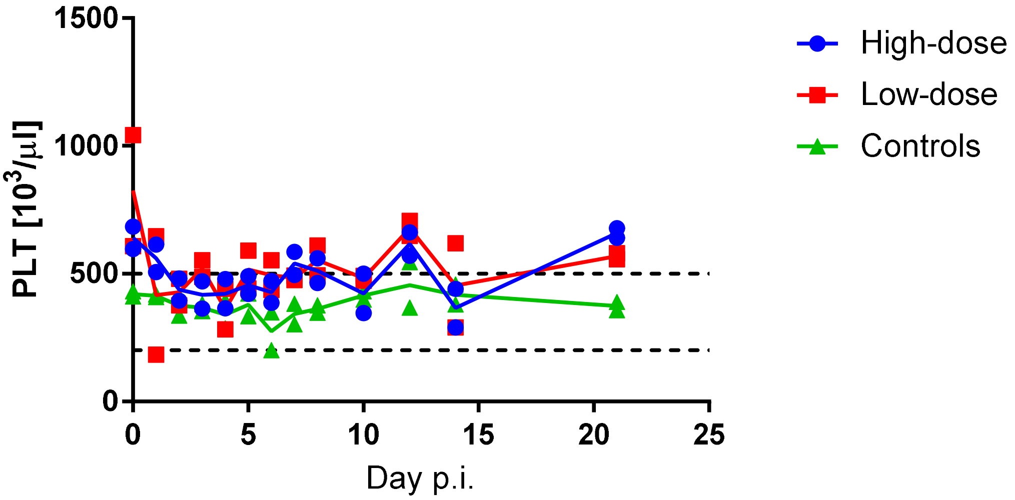

PLT [10^3/µl]
1500
1000
500
0
0
5
10
15
20
25
Day p.i.
High-dose
Low-dose
Controls

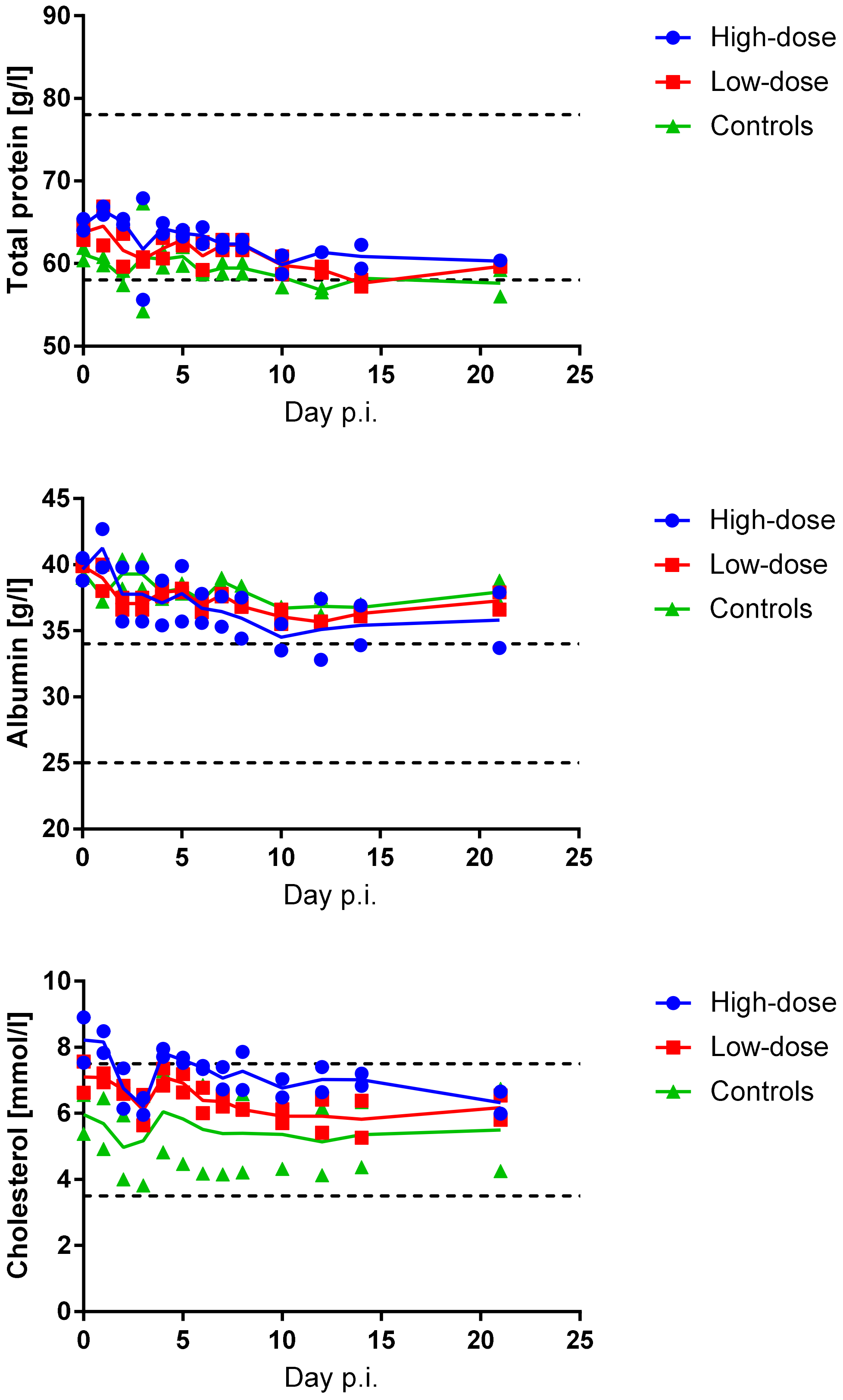

Total protein [g/l]
90
80
70
60
50
0
5
10
15
20
25
Day p.i.
High-dose
Low-dose
Controls
Albumin [g/l]
45
40
35
30
25
20
0
5
10
15
20
25
Day p.i.
High-dose
Low-dose
Controls
Cholesterol [mmol/l]
10
8
6
4
2
0
0
5
10
15
20
25
Day p.i.
High-dose
Low-dose
Controls

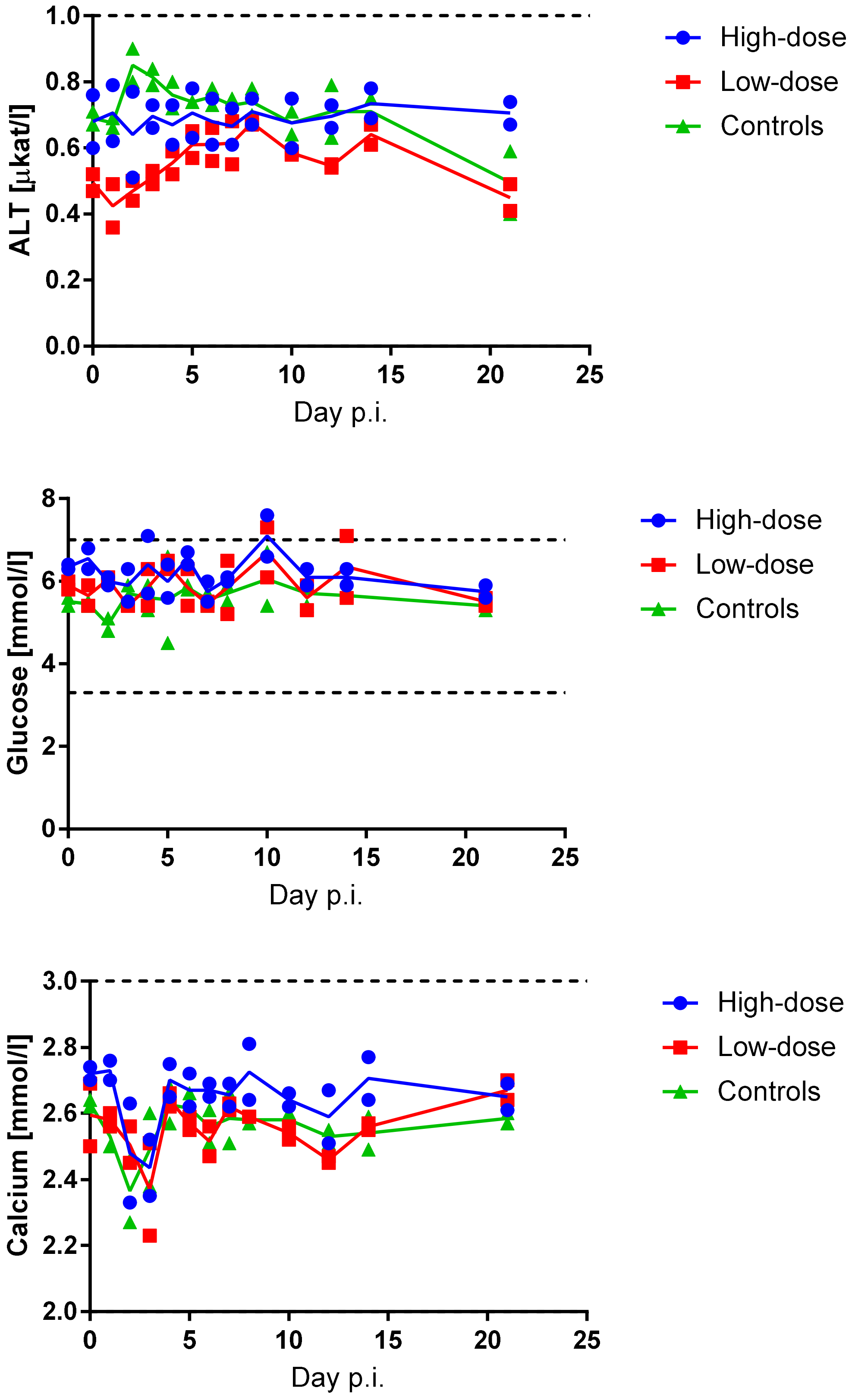
ALT [µkat/l]
1.0
0.8
0.6
0.4
0.2
0.0
0
5
10
15
20
25
Day p.i.
High-dose
Low-dose
Controls
Glucose [mmol/l]
8
6
4
2
0
0
5
10
15
20
25
Day p.i.
High-dose
Low-dose
Controls
Calcium [mmol/l]
3.0
2.8
2.6
2.4
2.2
2.0
0
5
10
15
20
25
Day p.i.
High-dose
Low-dose
Controls

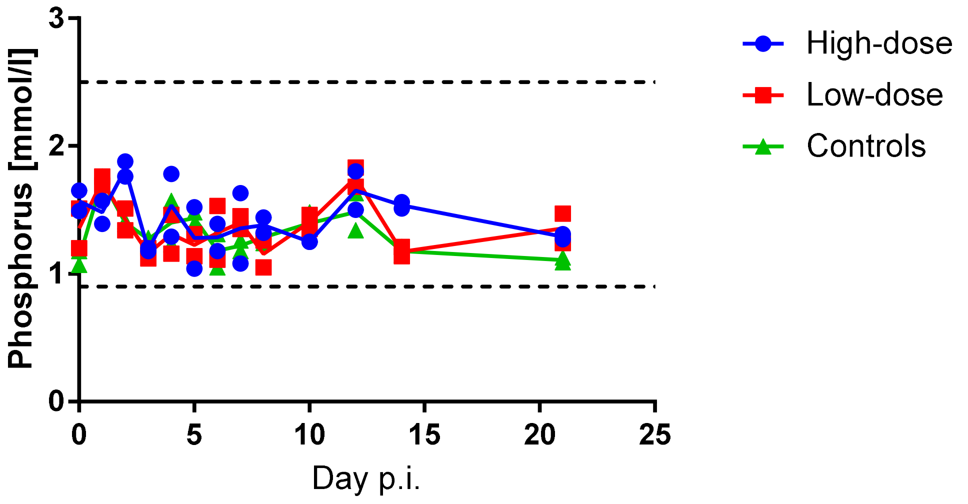
Phosphorus [mmol/l]
0
1
2
3
0
5
10
15
20
25
Day p.i.
High-dose
Low-dose
Controls

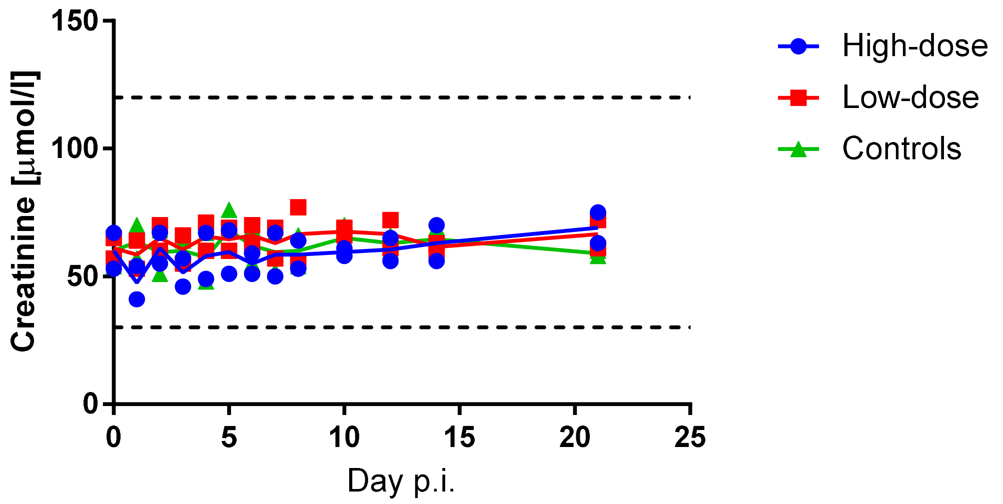
Creatinine [μmol/l]
0
50
100
150
0
5
10
15
20
25
Day p.i.
High-dose
Low-dose
Controls

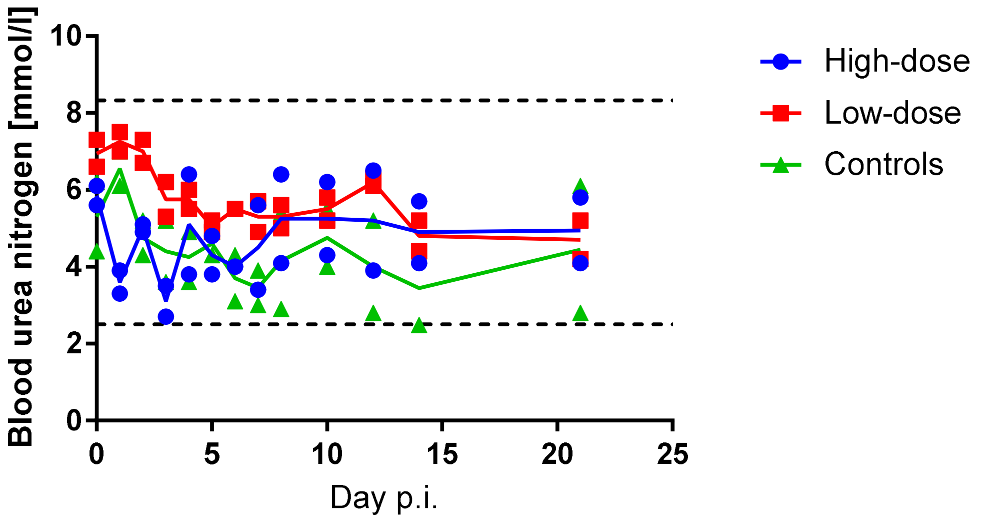
Blood urea nitrogen [mmol/l]
0
2
4
6
8
10
0
5
10
15
20
25
Day p.i.
High-dose
Low-dose
Controls
